# A systematic review and meta-analysis of perioperative oral decontamination in patients undergoing major elective surgery

**DOI:** 10.1186/s13741-016-0030-7

**Published:** 2016-03-22

**Authors:** Philip Spreadborough, Sarah Lort, Sandro Pasquali, Matthew Popplewell, Andrew Owen, Irene Kreis, Olga Tucker, Ravinder S Vohra, on behalf of the Preventing Postoperative Pneumonia Study Group and the West Midlands Research Collaborative

**Affiliations:** West Midlands Research Collaborative, University of Birmingham, Edgbaston, Birmingham, B15 2TH UK; Department of Upper Gastro-Intestinal Surgery, Queen Elizabeth Hospital, Birmingham, UK; School of Immunity and Infection, University of Birmingham, Birmingham, UK; Clinical Effectiveness Unit, Royal College of Surgeons England, London, UK; Academic Department of Surgery, University of Birmingham, 4th Floor, (Old) Queen Elizabeth Hospital, Edgbaston, Birmingham, B15 2TH UK; Nottingham Oesophagi-Gastric unit, Nottingham University Hospitals NHS Trust, Queens Medical Centre, Nottingham, NG7 2UH UK

**Keywords:** Anti-infective agents, Chlorhexidine, Perioperative care, Pneumonia

## Abstract

**Background:**

Oral antiseptics reduce nosocomial infections and ventilator-associated pneumonia in critically ill medical and surgical patients intubated for prolonged periods. However, the role of oral antiseptics given before and after planned surgery is not clear. The aim of this systematic review and meta-analysis is to determine the effect of oral antiseptics (chlorhexidine or povidone–iodine) when administered before and after major elective surgery.

**Methods:**

Searches were conducted of the MEDLINE, EMBASE and Cochrane databases. The analysis was performed using the random-effects method and the risk ratio (RR) with 95 % confidence interval (CI).

**Results:**

Of 1114 unique identified articles, perioperative chlorhexidine was administered to patients undergoing elective surgery in four studies. This identified 2265 patients undergoing elective cardiac surgery, of whom 1093 (48.3 %) received perioperative chlorhexidine. Postoperative pneumonia and nosocomial infections were observed in 5.3 and 20.2 % who received chlorhexidine compared to 10.4 and 31.3 % who received a control preparation, respectively. Oral perioperative chlorhexidine significantly reduced the risk of postoperative pneumonia (RR = 0.52; 95 % CI 0.39–0.71; *p* < 0.01) and overall nosocomial infections (RR = 0.65; 95 % CI 0.52–0.81; *p* < 0.01), with no effect on in-hospital mortality (RR = 1.01; 95 % CI 0.49–2.09; *p* = 0.98).

**Conclusions:**

Perioperative oral chlorhexidine significantly decreases the incidence of nosocomial infection and postoperative pneumonia in patients undergoing elective cardiac surgery. There are no randomised controlled studies of this simple and cheap intervention in patients undergoing elective non-cardiac surgery.

**Trial Registration:**

This systematic review was registered with the International prospective register of systematic reviews (PROSPERO). The registration number is CRD42015016063.

## Background

An estimated 234 million patients undergo major surgery worldwide every year. Nosocomial infections, particularly postoperative pneumonia, following surgery are common, affecting 1.5–57 % of patients depending on the type and extent of surgery (Weiser et al. 2008; Hemmes et al. 2014; Niggebrugge et al. 1999; Treschan et al. 2012; Seiler et al. 2009; Hulscher et al. 2002). Following major elective abdominal surgery, postoperative pneumonia results in six to nine extra hospital days and costs the healthcare system an additional $30,000 per patient (Khuri et al. 2005). Even after risk adjustment, it is associated with a 66 % lower survival at 5 years following surgery (Khuri et al. 2005). In those who do survive, the limited available evidence suggests a detrimental effect on early and late health-related quality of life (Thompson et al. 2006).

The definition of postoperative pneumonia used in the majority of studies is based on clinical, radiological and microbiological criteria defined by the Center of Disease Control and Prevention (CDC) for nosocomial pneumonia between the 2^nd^ and 30^th^ postoperative days (Garner et al. 1988). One of the primary causes of postoperative pneumonia is aspiration of oral and pharyngeal secretions at the time of intubation before surgery. Continued micro-aspiration of secretions due to small folds in the endotracheal tube cuff with prolonged ventilation (days to weeks) contributes to ventilator-associated pneumonia (VAP) (du Moulin et al. 1982; Cook et al. 1998; American Thoracic Society 2005). Oral antiseptics such as chlorhexidine gluconate or povidone–iodine have been shown to reduce the oral bacterial load in patients mechanically ventilated for 3 days or more. Chlorhexidine gluconate is a broad-spectrum antimicrobial, effective against gram-positive and gram-negative bacteria, anaerobes and fungi within 20 s (Horner et al. 2012; Fitzgerald et al. 1989). Three recent systematic reviews demonstrate reduction in VAP by 20 % with regular oral chlorhexidine application after intubation in critically ill patients mechanically ventilated for 3 days or more, with conflicting effects on early mortality (Labeau et al. 2011; Klompas et al. 2014; Price et al. 2014). Recent recommendations support daily chlorhexidine mouth care to prevent VAP in the intensive care setting (Scottish Intensive Care Society Audit Group 2008). However, the majority of elective surgical patients are extubated immediately following surgery in the operating room. These recommendations of daily chlorhexidine mouth care do not apply to this group, and pre-anaesthesia oral decontamination or prophylaxis with oral antiseptics is currently not part of the routine care. The aim of this systematic review and meta-analysis is to determine the effect of oral decontamination using antiseptics (chlorhexidine or povidone–iodine) before and after major elective surgery on infective complications and postoperative mortality.

## Methods

### Study selection

The meta-analysis was performed following the Preferred Reporting Items for Systematic Reviews and Meta-Analyses (PRISMA) guidelines (Moher et al. 2009). A systematic review was conducted by searching the MEDLINE, EMBASE and Cochrane databases. The full search criteria used are included at Appendix A. They contain search terms used relating to “surgery” and any combination of “chlorhexidine”, “iodine”, “povidone” with terms relating to “mouth”, “oral” and “decontamination”. This was limited to a 20-year period between October 1994 and 2014 and English language publications. All trial designs and interventions (mouthwash, nasal, gel) were included. Studies in patients under 18 years and including dental, oral or maxillofacial surgery were excluded.

### Data extraction and synthesis

Two investigators independently reviewed the search results. A third investigator resolved any disagreements. Two additional investigators assessed all included papers. The perioperative period was defined as any time period before and after the operation. Risk of bias was assessed using the Cochrane Collaboration checklist and the Jadad score (Jadad et al. 1996).

### Main outcomes and measures

Outcomes assessed were postoperative pneumonia and overall nosocomial infections, mortality, and intervention-related adverse events. Postoperative pneumonia was defined as nosocomial pneumonia between the 2nd and 30^th^ postoperative days based on the CDC criteria (Garner et al. 1988). Nosocomial infections were defined as surgical site infections and any other infections including postoperative pneumonia, urinary tract infections and bacteraemia between the 2^nd^ and 30^th^ postoperative days (Garner et al. 1988). The additional following information was sought from all the included papers: study design, eligibility criteria, randomization method, allocation method, risk category, strength of solutions used, treatment regime and number of randomised patients.

### Statistical analysis

A meta-analysis methodology was applied to determine the effect of a perioperative oral antiseptic on the incidence of postoperative pneumonia, nosocomial infections and mortality following surgery (Higgins and Green 2011). Data were analysed on an intention-to-treat principle. When this information was not available, per-protocol data were used. The outcome measures were the risk ratio (RR), with 95 % confidence interval (CI), weighted by the inverse of their variances. In this meta-analysis, mouthwash is considered the “experimental” treatment with RR reported as mouth wash-to-placebo/observation ratios.

We assessed heterogeneity using chi^2^-based Cochran’s *Q* test and *I*^2^ statistic tests. Inconsistency across studies was considered low, moderate and high for *I*^2^ statistic values lower than 25 %, between 25 and 50 % and greater than 50 %, respectively. Heterogeneity was significant when the *I*^2^ statistic was greater than 50 %, the Cochran’s *Q* test *p* value was smaller than 0.1 or both. A random-effects model was used to calculate the overall effect.

## Results

**Fig. 1:**
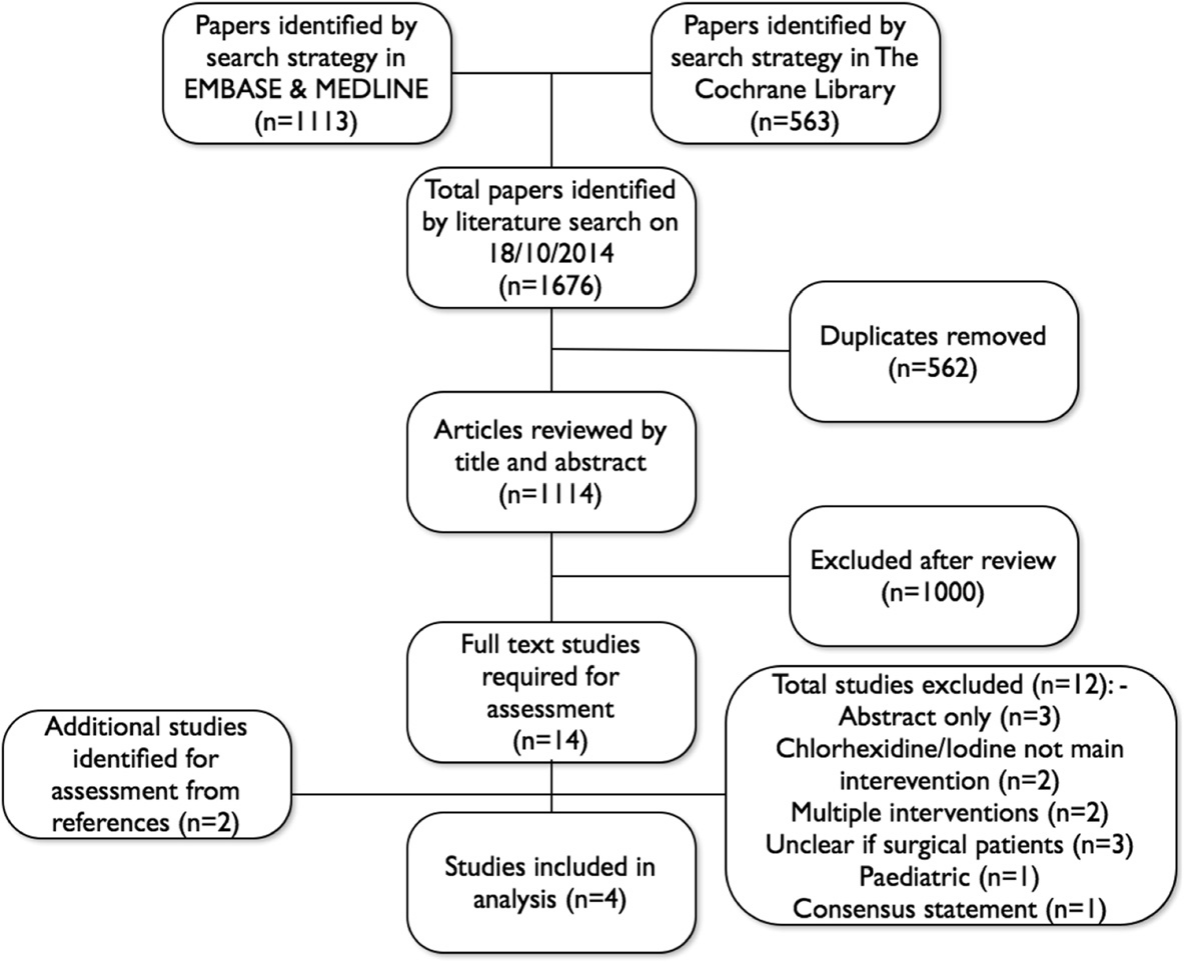
CONSORT flow diagram of articles included in the systematic review

One thousand six hundred seventy-six articles were identified (Fig. 1). Five hundred sixty-two duplicates and a further 1100 were excluded after abstract review. Full text was not available for 3 of the 14 remaining abstracts. Four of the 11 publications met the criteria after full manuscript review. These studies included 2205 participants of whom 1093 received perioperative chlorhexidine mouthwash or gel. None of the studies reported iodine use. All four studies, three randomised controlled and one quasi-experimental, included patients having elective cardiac surgery only. Table 1 summarises the sample sizes, population, intervention regime and outcomes of the eligible studies. Additional preparations were administered in two studies with nasal chlorhexidine gel in one, and dental brushing in another. All four studies included a placebo (mouthwash, gel or nasal ointment).

**Table 1:** Summary of sample sizes, population, regime, and outcomes

Author	Location	Patients (chlorhexidine vs. control)	Population	Chlorhexidine strength	Regime	Overall nosocomial infection	Postoperative pneumonia	Mortality
De Riso 1996	USA	353 (173 vs. 180)	Cardiac	0.12 %	Preop (no time scale given) and postop (discharge from ITU or death). Mean = 8.2 days	8/173 (4.6 %) 24/180 (13.3 %)	5/173 (2.9 %) 17/180 (9.4 %)	3/173 (1.7 %) 10/180 (5.6 %)
Houston et al 2002	USA	561 (270 vs. 291)	Cardiac	0.12 %	Preop (no time scale given) and postop (10 days or extubation, tracheostomy, development of POP or death)	–	4/270 (1.5 %) 9/291 (3.1 %)	6/270 (2.2 %) 3/291 (1 %)
Nicolosi et al 2014	Argentina	300 (150 vs. 150)	Cardiac	0.12 %	Preop (3 days)	46/150 (30.7 %) 69/150 (46 %)	4/150 (2.7 %) 13/150 (8.7 %)	8/150 (5.3 %) 7/150 (4.7 %)
Segers et al. 2006	USA	991 (500 vs. 491)	Cardiac	0.12 %	Preop (mean = 1.9 days) and postop (no time scale given)	116/500 (23.2 %) 164/491 (33.4 %)	45/500 (9 %) 74/491 (15.1 %)	8/500 (1.6 %) 6/491 (1.2 %)

All four studies reported postoperative pneumonia rates and mortality, while three reported nosocomial infection rates (Segers et al. 2006; DeRiso et al. 1996; Nicolosi et al. 2014). Three studies used intention-to-treat analysis (DeRiso et al. 1996; Nicolosi et al. 2014; Houston et al. 2002), and one a *per-protocol* analysis (Segers et al. 2006). The risk of bias and Jadad scores are summarised in Table 2. The chlorhexidine regime used varied. All four studies included preoperative chlorhexidine. Three studies continued the intervention postoperatively with varying duration and preparations (Segers et al. 2006; DeRiso et al. 1996; Houston et al. 2002). Only one study reported duration and dosing (Nicolosi et al. 2014).

**Table 2:** Risk of bias in studies

Study	Random sequence generation	Allocation concealment	Blinding	Incomplete data outcome addressed
Nicolosi et al. 2014	N/A	N/A	N/A	N/A
Segers et al. 2006	Low risk	Low risk	Low risk	Low risk
Houston et al. 2002	High risk	Unclear	Unclear	Low risk
DeRiso et al. 1996	Low risk	Low risk	Low risk	Low risk

*N/A* not applicable

### Postoperative pneumonia

**Fig. 2:**
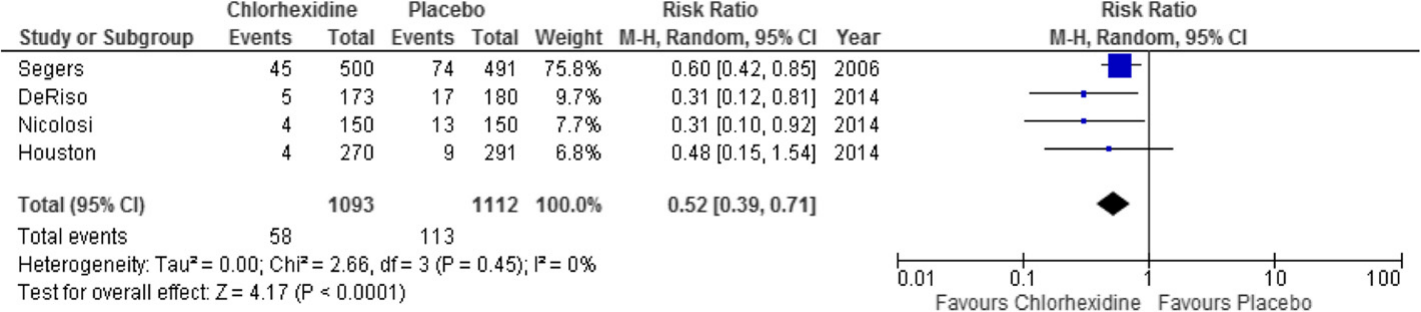
*Forest plot* comparing postoperative pneumonia in patients with or without chlorhexidine cover. A Mantel–Haenszel random-effects model was used for meta-analysis. Risk ratios are shown with 95 % confidence interval

Three of the four studies used the CDC definition (American Thoracic Society 2005; Segers et al. 2006; DeRiso et al. 1996; Houston et al. 2002; Rotstein et al. 2008). Timing of the diagnosis was variable and not reported in one study (Segers et al. 2006; DeRiso et al. 1996; Nicolosi et al. 2014; Houston et al. 2002). Fifty-eight (5.3 %) patients in the chlorhexidine group developed postoperative pneumonia compared with 113 (10.2 %) patients in the control group (RR = 0.52; 95 % CI 0.39–0.71; *p* < 0.01). There was no statistical significant between study heterogeneity (*p* = 0.45; *I*^2^ = 0 %). This produced a number needed to treat of 14 (Fig. 2).

### Nosocomial infections

**Fig. 3:**
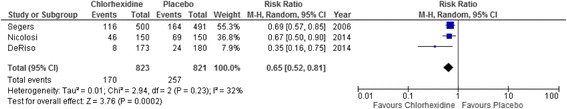
*Forest plot* comparing nosocomial infections in patients with or without chlorhexidine cover. A Mantel–Haenszel random-effects model was used for meta-analysis. Risk ratios are shown with 95 % confidence interval

Of the three studies that reported nosocomial infection rates, 170 (20.7 %) patients in the chlorhexidine group, compared with 265 (31.3 %) in the control arm, developed a nosocomial infection (RR = 0.65; 95 % CI 0.52–0.81; *p* < 0.01). There was no statistical significant between study heterogeneity (*p* = 0.23; *I*^2^ = 32 %). This produced a number needed to treat of 9 (Fig. 3).

### Mortality

**Fig. 4:**
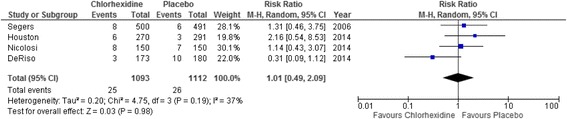
Forest plot comparing in-hospital mortality in patients with or without chlorhexidine cover. A Mantel–Haenszel random-effects model was used for meta-analysis. Risk ratios are shown with 95 % confidence interval

 All four studies reported in-hospital mortality (Fig. 4) with 25 (2.3 %) deaths in the chlorhexidine group compared with 26 (2.3 %) in the control arm (RR = 1.01; 95 % CI 0.49–2.09; *p* = 0.98). There was no statistical significant between study heterogeneity (*p* = 0.19; *I*^2^ = 37 %).

### Adverse events

Temporary teeth discolouration was reported in a study, in 1 of 500 patients (0.2 %) who received chlorhexidine.

## Discussion

Routine administration of oral chlorhexidine preparations before and after oral surgery reduces local and systemic infective complications (Berchier et al. 2010; Supranoto S et al. 2015; Lambert et al. 1997). This is the first systematic review to determine the effectiveness of perioperative oral antiseptic use in patients undergoing major elective surgery. When administered both before and after elective cardiac surgery, oral antiseptic use with chlorhexidine significantly reduces the incidence of postoperative pneumonia and nosocomial infections with no effect on early mortality. The previous meta-analyses and current National Institute for Health and Care Excellence (NICE) and CDC guidelines focus on critically ill patients including emergency and elective surgical patients mechanically ventilated for 3 days or more (National Institute for Health and Care Excellence 2014; Tablan et al. 2004). The meta-analysis by Labeau et al. demonstrated a significant reduction in VAP (RR 0.67; 95 % CI 0.50–0.88; *p* < 0.01) with oral chlorhexidine or povidone–iodine use (Labeau et al. 2011). The effect was highest with chlorhexidine (RR 0.72; 95 % CI 0.55–0.94; *p* = 0.02). A subgroup analysis of two studies suggested patients undergoing cardiac surgery might have the greatest benefit from oral antiseptic use (RR 0.41; 95 % CI 0.17–0.98; *p* = 0.05) (Labeau et al. 2011).

This systematic review aimed to identify studies investigating the effectiveness of oral antiseptic use before and after major elective surgery. No studies were identified that included patients undergoing elective non-cardiac surgery or who were administered oral povidone–iodine.

Even though the number of studies performed is small, a clear benefit was demonstrated in the reduction of the incidence of postoperative pneumonia and nosocomial infection with perioperative oral chlorhexidine. Perioperative oral chlorhexidine would need to be administered to 14 patients to prevent one episode of postoperative pneumonia and 9 patients to prevent one episode of nosocomial infection. These small numbers needed to treat further supports the effectiveness of the intervention.

Other approaches, including selective decontamination of the digestive tract with antibiotics, reduce the incidence of postoperative pneumonia and nosocomial infection; however, uptake of this technique is limited by concerns of emerging antibiotic resistance (Silvestri and van Saene 2010; Nathens and Marshall 1999; Silvestri and van Saene 2012; Bastin and Ryanna 2009). These approaches were not considered in this systematic review. As the four studies identified included cardiac patients only the findings may not be generalisable to non-cardiac surgical cohorts.

Currently, oral chlorhexidine preparations are used to control dental plaque, treat gingivitis and given routinely before and after oral surgery to reduce local and systemic infective complications (Berchier et al. 2010; Supranoto et al. 2015; Lambert et al. 1997). Outside this setting, oral chlorhexidine preparations are not routinely administered in the perioperative setting. A recent international consensus statement from over 1000 anaesthetists, intensive care specialists, surgeons, and epidemiologists identified oral chlorhexidine preparations as an inexpensive intervention that may reduce perioperative mortality across surgical disciplines (Landoni et al. 2012). The expert panel commented that the lack of availability of effectiveness studies evaluating the use of perioperative chlorhexidine preparations has in part prevented its widespread adoption (Landoni et al. 2012; Rello et al. 2007).

Oral chlorhexidine is recommended in patients who remain intubated for prolonged periods as it is proven to reduce the incidence of VAP. Perioperative oral chlorhexidine reduces the incidence of postoperative pneumonia and nosocomial infections following elective cardiac surgery. No studies have been performed to evaluate the effectiveness of perioperative oral chlorhexidine on nosocomial infections and postoperative pneumonia after elective non-cardiac surgery. We suggest that a pragmatic, multi-centre and large clinical trial is needed to demonstrate the effectiveness of this simple, well-tolerated, and cheap intervention before and after elective major non-cardiac surgery before it will be accepted and introduced into complex perioperative clinical care pathways.

## Conclusions

Perioperative oral chlorhexidine significantly decreases the incidence of nosocomial infection and postoperative pneumonia in patients undergoing elective cardiac surgery. There are no randomised controlled studies of this simple and cheap intervention in patients undergoing elective non-cardiac surgery. Given the low number needed to treat to prevent either event, we suggest that this intervention may benefit patients undergoing major elective non-cardiac surgery, but additional research is required prior to its routine adoption in perioperative care pathways.

## Abbreviations

CDC: Center of Disease Control and Prevention
CI: confidence interval
*I*^2^: percentage variation across studies attributed to heterogeneity
NICE: National Institute for Health and Care Excellence
PRISMA: Preferred Reporting Items for Systematic Reviews and Meta-Analyses
RR: risk ratio
VAP: ventilator-associated pneumonia

## References

[CR1] Weiser TG, Regenbogen SE, Thompson KD, Haynes AB, Lipsitz SR, Berry WR (2008). An estimation of the global volume of surgery: a modelling strategy based on available data. Lancet.

[CR2] Hemmes SN, Gama de Abreu M, Pelosi P, Schultz MJ, PROVE Network Investigators for the Clinical Trial Network of the European Society of Anaesthesiology (2014). High versus low positive end-expiratory pressure during general anaesthesia for open abdominal surgery (PROVHILO trial): a multicentre randomised controlled trial. Lancet.

[CR3] Niggebrugge AH, Trimbos JB, Hermans J, Steup WH, Van De Velde CJ (1999). Influence of abdominal-wound closure technique on complications after surgery: a randomised study. Lancet.

[CR4] Treschan TA, Kaisers W, Schaefer MS, Bastin B, Schmalz U, Wania V (2012). Ventilation with low tidal volumes during upper abdominal surgery does not improve postoperative lung function. Br J Anaesth.

[CR5] Seiler CM, Deckert A, Diener MK, Knaebel HP, Weigand MA, Victor N (2009). Midline versus transverse incision in major abdominal surgery: a randomized, double-blind equivalence trial (POVATI: ISRCTN60734227). Ann Surg.

[CR6] Hulscher JB, van Sandick JW, de Boer AG, Wijnhoven BP, Tijssen JG, Fockens P (2002). Extended transthoracic resection compared with limited transhiatal resection for adenocarcinoma of the esophagus. N Engl J Med.

[CR7] Khuri SF, Henderson WG, DePalma RG, Mosca C, Healey NA, Kumbhani DJ (2005). Participants in the VANSQIP: determinants of long-term survival after major surgery and the adverse effect of postoperative complications. Ann Surg.

[CR8] Thompson DA, Makary MA, Dorman T, Pronovost PJ (2006). Clinical and economic outcomes of hospital acquired pneumonia in intra-abdominal surgery patients. Ann Surg.

[CR9] Garner JS, Jarvis WR, Emori TG, Horan TC, Hughes JM (1988). CDC definitions for nosocomial infections, 1988. Am J Infect Control.

[CR10] du Moulin GC, Paterson DG, Hedley-Whyte J, Lisbon A (1982). Aspiration of gastric bacteria in antacid-treated patients: a frequent cause of postoperative colonisation of the airway. Lancet.

[CR11] Cook D, De Jonghe B, Brochard L, Brun-Buisson C (1998). Influence of airway management on ventilator-associated pneumonia: evidence from randomized trials. JAMA.

[CR12] American Thoracic Society, Infectious Diseases Society of America (2005). Guidelines for the management of adults with hospital-acquired, ventilator-associated, and healthcare-associated pneumonia. Am J Respir Crit Care Med.

[CR13] Horner C, Mawer D, Wilcox M (2012). Reduced susceptibility to chlorhexidine in staphylococci: is it increasing and does it matter?. J Antimicrob Chemother.

[CR14] Fitzgerald KA, Davies A, Russell AD (1989). Uptake of 14C-chlorhexidine diacetate to Escherichia coli and Pseudomonas aeruginosa and its release by azolectin. FEMS Microbiol Lett.

[CR15] Labeau SO, Van de Vyver K, Brusselaers N, Vogelaers D, Blot SI (2011). Prevention of ventilator-associated pneumonia with oral antiseptics: a systematic review and meta-analysis. Lancet Infect Dis.

[CR16] Klompas M, Speck K, Howell MD, Greene LR, Berenholtz SM (2014). Reappraisal of routine oral care with chlorhexidine gluconate for patients receiving mechanical ventilation: systematic review and meta-analysis. JAMA Intern Med.

[CR17] Price R, MacLennan G, Glen J, Su DC (2014). Selective digestive or oropharyngeal decontamination and topical oropharyngeal chlorhexidine for prevention of death in general intensive care: systematic review and network meta-analysis. BMJ.

[CR18] Scottish Intensive Care Society Audit Group: VAP prevention bundle: guidance for implementation. National Health Services Scotland 2008.

[CR19] Moher D, Liberati A, Tetzlaff J, Altman DG, Group P (2009). Preferred Reporting Items for Systematic Reviews and Meta-Analyses: the PRISMA statement. BMJ.

[CR20] Jadad AR, Moore RA, Carroll D, Jenkinson C, Reynolds DJ, Gavaghan DJ (1996). Assessing the quality of reports of randomized clinical trials: is blinding necessary?. Control Clin Trials.

[CR21] Higgins JPT, Green S (editors): Cochrane handbook for systematic reviews of interventions version 5.1.0. March 2011 edition: The Cochrane Collaboration; 2011. Available from www.cochranehandbook.org.

[CR22] Segers P, Speekenbrink RG, Ubbink DT, Ogtrop ML, Mol BA (2006). Prevention of nosocomial infection in cardiac surgery by decontamination of the nasopharynx and oropharynx with chlorhexidine gluconate: a randomized controlled trial. JAMA.

[CR23] DeRiso AJ, Ladowski JS, Dillon TA, Justice JW, Peterson AC (1996). Chlorhexidine gluconate 0.12 % oral rinse reduces the incidence of total nosocomial respiratory infection and nonprophylactic systemic antibiotic use in patients undergoing heart surgery. Chest.

[CR24] Nicolosi LN, del Carmen Rubio M, Martinez CD, Gonzalez NN, Cruz ME (2014). Effect of oral hygiene and 0.12 % chlorhexidine gluconate oral rinse in preventing ventilator-associated pneumonia after cardiovascular surgery. Respir Care.

[CR25] Houston S, Hougland P, Anderson JJ, LaRocco M, Kennedy V, Gentry LO (2002). Effectiveness of 0.12 % chlorhexidine gluconate oral rinse in reducing prevalence of nosocomial pneumonia in patients undergoing heart surgery. Am J Crit Care.

[CR26] Rotstein C, Evans G, Born A, Grossman R, Light RB, Magder S (2008). Clinical practice guidelines for hospital-acquired pneumonia and ventilator-associated pneumonia in adults. Can J Infect Dis Med Microbiol.

[CR27] Berchier CE, Slot DE, Van der Weijden GA (2010). The efficacy of 0.12 % chlorhexidine mouthrinse compared with 0.2 % on plaque accumulation and periodontal parameters: a systematic review. J Clin Periodontol.

[CR28] Supranoto S, Slot D, Addy M, Van der Weijden G: The effect of chlorhexidine dentifrice or gel versus chlorhexidine mouthwash on plaque, gingivitis, bleeding and tooth discoloration: a systematic review. Int J Dent Hyg 2015;13(2):83-92. 10.1111/idh.12078 25059640

[CR29] Lambert PM, Morris HF, Ochi S (1997). The influence of 0.12 % chlorhexidine digluconate rinses on the incidence of infectious complications and implant success. J Oral Maxillofac Surg.

[CR30] National Institute for Health and Care Excellence (2014). Technical patient safety solutions for ventilator-associated pneumonia in adults.

[CR31] Tablan OC, Anderson LJ, Besser R, Bridges C, Hajjeh R (2004). CDC, Healthcare Infection Control Practices Advisory C: Guidelines for preventing health-care-associated pneumonia, 2003: recommendations of CDC and the Healthcare Infection Control Practices Advisory Committee. MMWR Recomm Rep.

[CR32] Silvestri L, van Saene HK (2010). Selective digestive decontamination to prevent pneumonia after esophageal surgery. Ann Thorac Cardiovasc Surg.

[CR33] Nathens AB, Marshall JC (1999). Selective decontamination of the digestive tract in surgical patients: a systematic review of the evidence. Arch Surg.

[CR34] Silvestri L, van Saene HK (2012). Selective decontamination of the digestive tract: an update of the evidence. HSR Proc Intensive Care Cardiovasc Anesth.

[CR35] Bastin AJ, Ryanna KB (2009). Use of selective decontamination of the digestive tract in United Kingdom intensive care units. Anaesthesia.

[CR36] Landoni G, Rodseth RN, Santini F, Ponschab M, Ruggeri L, Szekely A (2012). Randomized evidence for reduction of perioperative mortality. J Cardiothorac Vasc Anesth.

[CR37] Rello J, Koulenti D, Blot S, Sierra R, Diaz E, De Waele JJ (2007). Oral care practices in intensive care units: a survey of 59 European ICUs. Intensive Care Med.

